# Identifying what matters to adults with mobility limitations regarding their experiences with medications: A concept mapping study

**DOI:** 10.1371/journal.pone.0323877

**Published:** 2025-05-23

**Authors:** Sara J. T. Guilcher, Lauren Cadel, Amanda C. Everall, Anita Kaiser, Stephanie R. Cimino, Rasha El-Kotob, Lisa McCarthy, Colleen O’Connell, Crystal MacKay, James Milligan, Aisha Lofters, Sander L. Hitzig, Diana Zidarov

**Affiliations:** 1 Leslie Dan Faculty of Pharmacy, University of Toronto, Toronto, Ontario, Canada; 2 St. John’s Rehab Research Program, Sunnybrook Research Institute, Sunnybrook Health Sciences Centre, Toronto, Ontario, Canada; 3 Rehabilitation Sciences Institute, Temerty Faculty of Medicine, University of Toronto, Toronto, Ontario, Canada; 4 Department of Physical Therapy, Temerty Faculty of Medicine, University of Toronto, Toronto, Ontario, Canada; 5 Institute for Better Health, Trillium Health Partners, Mississauga, Ontario, Canada; 6 Canadian Spinal Research Organization, Richmond Hill, Ontario, Canada; 7 KITE, Toronto Rehab-University Health Network, Toronto, Ontario, Canada; 8 School of Rehabilitation Therapy, Queen’s University, Kingston, Ontario, Canada; 9 Department of Medicine, Dalhousie University, Halifax, Nova Scotia, Canada; 10 West Park Healthcare Centre, Toronto, Ontario, Canada; 11 Mobility Clinic at the Centre for Family Medicine, Kitchener-Waterloo, Ontario, Canada; 12 Peter Gilgan Centre for Women’s Cancers, Women’s College Hospital, Toronto, Ontario, Canada; 13 Department of Occupational Science and Occupational Therapy, Temerty Faculty of Medicine, University of Toronto, Toronto, Ontario, Canada; 14 Dalla Lana School of Public Health, University of Toronto, Toronto, Ontario, Canada; 15 Institut universitaire sur la réadaptation en déficience physique de Montréal, Centre intégré universitaire de santé et de services sociaux du Centre-Sud-de-l’Île-de-Montréal, Montreal, Quebec, Canada; 16 Centre de Recherche Interdisciplinaire en Réadaptation du Montréal Métropolitain, Montreal, Quebec, Canada; Birjand University of Medical Sciences, ISLAMIC REPUBLIC OF IRAN

## Abstract

**Background:**

Despite the high prevalence of medication use among persons with mobility limitations, there are currently no patient-reported measures that have been co-developed to assess the experiences of medications in everyday life. Therefore, the objective of this study was to develop potential items for a patient-reported experience measure related to medication use for adults with mobility limitations.

**Methods:**

We conducted a concept mapping study with people with mobility limitations. Participants were required to: be 18 years of age or older, live in Canada, live in the community, speak, and read English or French, have a mobility limitation, and take at least one medication recommended by a prescriber in the preceding three months. Participants generated statements in response to the focal prompt: what matters to you about medications in your everyday life? Participants then sorted piles of statements based on their conceptual similarity, rated each statement on two dimensions (importance and realistic), and created visual maps of the data.

**Results:**

A total of 45 individuals participated in at least one step of the concept mapping. Participants generated 694 statements which were synthesized into 80 unique statements. The final map contained ten clusters: (1) medication-related financial considerations and support; (2) pharmacy-related services and supports; (3) access to medications and medication-related supports; (4) acceptance and stigma around medication use; (5) ability and ease of taking medications; (6) shared decision-making and access to medication-related research and information; (7) medication effectiveness, side effects and risks; (8) knowledge, self-awareness and empowerment; (9) accessibility of healthcare providers; and (10) communication and relationships with healthcare providers.

**Conclusions:**

In this participatory-based research, we have identified key items and domains related to medication-related experiences. Understanding what matters to patients will support quality improvement of healthcare delivery and outcomes for adults with mobility limitations who take medications.

## Introduction

In Canada, there are approximately 8 million individuals 15 years or older with a self-reported disability [1]. Over a third of these individuals have mobility limitations, with a prevalence of 39.2% [1]. Persons with mobility limitations often experience multiple chronic conditions [2], which may be treated with medications [3–5]. The use of multiple medications, known as polypharmacy (five or more medications), can increase the risk of adverse medical outcomes and experiences [6]. Problematic polypharmacy has been linked to an increase in adverse drug events (e.g., falls, confusion) and mortality [7]. Impaired physical function can increase the likelihood of polypharmacy, which can then negatively impact physical function (e.g., walking speed, standing balance) [8].

The World Health Organization has identified ‘Medications without Harm’ as the third global patient safety challenge and has established priority approaches for improving medication safety, such as ongoing monitoring of experiences and patient engagement [9]. Aligned with the Quintuple Aim for value-based quality care [10], engaging persons with lived experience as active partners in care is part of a wider transformational paradigm shift in healthcare system performance; moreover, this shift recognizes the value of measuring patient perspectives on their experiences and outcomes [11,12]. Patient reported outcome measures (PROMs) are standardized and validated instruments that are directly reported by patients and relate to health, functional status, or quality of life [13]. Relatedly, patient reported experience measures (PREMs) capture patients’ experiences with care or treatments [14–16]. Interestingly, despite the high prevalence of medication use, to our knowledge, there are currently no patient-reported experience or outcome measures that are co-developed to measure and monitor the experiences of medications in everyday life for persons with mobility limitations [17]. Co-development supports meaningful engagement of the end users to help make research more useful for the intended population [18]. Therefore, co-development of PREMs would focus on what is important to people with mobility limitations.

Monitoring experiences and outcomes related to medications involves understanding impacts on multiple aspects of individuals’ day-to-day lives, including physical, mental, emotional, and social well-being [19]. In previous research, unique challenges were identified among persons with mobility limitations [20]. Individuals described anxiety regarding the short and long-term safety of medications, in addition to fears about medication effectiveness plateauing over time [20,21]. Further concerns were raised around medication side effects (e.g., fatigue, addiction, constipation) [20, 22] and regimen complexity. Individuals identified challenges with remembering and obtaining refills, remembering when to take medications, medication-related costs, and taking medications due to physical impairments and/or lack of physical and emotional support [20–24]. Importantly, people with mobility limitations experience unique barriers to services, including challenges with physical accessibility, transportation, attitudes and knowledge, social, and discriminatory policies [25–27].

Given the unique challenges that individuals with mobility limitations may experience in accessing healthcare and their medications, it is important that measures are developed with and/or validated among persons with these lived experiences. Systematically measuring what matters to individuals about medication use and how medications impact everyday life will ideally contribute to improved shared decision-making between healthcare providers and patients and safer care plans [28–30]. To address this gap, the objective of this study was to develop potential items for a PREM related to medication use for French and English-speaking persons with mobility limitations.

## Methods

### Study design

We took a participatory, mixed methods approach by applying concept mapping methodology [31]. Concept mapping is ideal for gathering perspectives from many individuals, with a focus on breadth of experiences rather than depth [32]. It is often used within public health for planning and evaluation purposes [32,33] and can be a useful way of conducting needs assessments of a target population [34]. Concept mapping involves six steps: preparation, brainstorming, sorting and rating, analysis, mapping and interpretation, and utilization.

This study received human ethics approvals from the University of Toronto, #42514 and the University of Montreal, #2022–1598. All participants provided written or verbal informed consent prior to participation. We allowed for verbal consent from participants who were unable to complete the written-and-signed consent approach due to functional limitations. For individuals consenting verbally, a consent form was sent to potential participants by email. A member of the research team connected with the potential participant by telephone or teleconference to review the consent form, answer questions, and obtain consent. Verbal consent was audio-recorded and tracked in a master log. The research ethics boards approved of this process.

### Step 1 – Preparation

#### Participants and recruitment.

To participate in the study, individuals were required to live in Canada, be 18 years of age or older, live in the community (i.e., not in a long-term care home or acute hospital), speak and read English or French, self-identify as having a mobility limitation, and take at least one medication recommended by a prescriber in the preceding three months. A mobility limitation was defined according to the World Health Organization Disability Assessment Schedule (WHODAS) 2.0 [35], where participants were required to have at least mild difficulty with one of the following: standing for long periods of time (e.g., 30 minutes), standing from sitting down, moving around inside the home, getting out of the home, and walking a long distance (e.g., one kilometer).

We recruited individuals with mobility limitations from across Canada using a multi-pronged approach. Participants were recruited between June 2022 and July 2023. Study recruitment flyers were placed in clinics that provided care to persons with mobility limitations, as well as in local community centers. Similarly, study advertisements were shared on social media, with a focus on social media sites for individuals with mobility limitations. We also recruited through the newsletters, websites, and listservs of Canadian organizations who have likely served populations with mobility limitations. Some individuals in the province of Quebec were identified through a clinical research database and were contacted by the research team directly.

### Step 2 – Brainstorming

The following focal prompt (main research question) was used to elicit responses from participants during the brainstorming sessions: *what matters to you about medications in your everyday life?* Participants generated ideas in response to the focal prompt through virtual focus groups, interviews, and groupwisdom™ (online concept mapping platform). The focus groups and interviews were approximately one hour in length and were conducted between June 2022 and September 2022 by three trained qualitative researchers (ACE, LC, JW). All focus groups were conducted in English, while interviews were conducted in both English and French due to team experience and participant capacity. As participants shared their thoughts, the statements were documented. All brainstorming sessions were audio recorded to ensure all statements were captured.

All participants completed a comprehensive questionnaire covering their demographics and health history, including type and number of chronic diseases, type and frequency of medications taken, and the impact that their mobility limitation had on their everyday life. English-speaking participants completed the questionnaire online through REDCap, while French-speaking participants were administered the questionnaire verbally at the time of interviews.

Following the brainstorming sessions, the research team engaged in the statement synthesis process to reduce the total number of ideas generated into a manageable number (e.g., less than 100 statements) for the sorting and rating tasks [32]. The team did so by removing statements that did not answer the focal prompt, combining similar ideas, and removing duplicate statements. All statements in the final list were reviewed for clarity by the broader research team. When uploaded to groupwisdom™, the statements were randomized and assigned a number for the sorting and rating tasks.

### Step 3 – Sorting and rating

The sorting and rating tasks were completed independently by participants on groupwisdom™. During the sorting task, participants created groups or piles of statements based on their conceptual similarity and assigned a title to each pile. Participants were instructed to group statements based on the following guidelines: (1) each statement can only be put into one pile; (2) each pile must contain at least two statements; (3) there must be more than one pile; and (4) piles must be created based on the content of the statement and not on a value judgement (e.g., piles should not be sorted based on importance). During the rating task, participants rated each statement from one to five on two Likert-type scales based on two domains: perceived importance and realistic (1 = not at all important/realistic to 5 = extremely important/realistic).

### Step 4 – Analysis

Analyses were conducted using groupwisdom™ to create visual maps for the mapping and interpretation session. A total square similarity matrix was created by combining similarity matrices. This displayed the number of participants who sorted each pair of statements together [36]. The total square similarity matrix was the input for multidimensional scaling, producing the point map (see S1 Fig). The stress value indicates the goodness of fit between the similarity matrix data and the point map. The calculated stress value was 0.25, which falls between the acceptable range for concept mapping projects of 0.205 and 0.365 [36]. Hierarchical cluster analysis was conducted to create groups of statements based on their relative distance. Prior to the mapping session, cluster map solutions were reviewed by the research team and two cluster maps were selected to present to the participants. The research team also reviewed the statements contained within each cluster and assigned a title to each.

### Step 5 – Mapping and interpretation

Participants attended a 90-minute virtual mapping session, where the goals were to select a cluster solution and finalize the statements within each cluster. The session was facilitated by a trained research team member (RE). Participants were shown two cluster map solutions, the 10-cluster map and the 11-cluster map. Once a final cluster solution was selected, the statements within each cluster were reviewed and participants had the opportunity to move statements to a different cluster where they thought they fit better based on concepts. The group achieved consensus through discussion prior to moving any statements.

After the mapping session, the research team created additional maps and visual representations of the data using groupwisdom™. Point rating maps, cluster rating maps, pattern match diagrams, and go-zone plots were developed to explore potential differences in the rating data based on participant demographics (gender, number of medications, age, type of mobility limitation, and difficulty walking).

### Step 6 – Utilization

The results from this concept mapping study will inform items to consider in a PREM. In consultation with a working group, the research team will apply for future funding to further develop a core set of items to include in a measure.

## Results

### Participant demographics

Twenty-two individuals participated in brainstorming, 30 completed sorting, 45 completed rating, and 9 participated in the mapping session (see Table 1 for participant demographics by each step). Some individuals participated in multiple steps of concept mapping. Across all concept mapping steps, most participants identified as women, experienced polypharmacy (the use of five or more medications), and had at least “moderate” difficulty walking a long distance. Variation was seen in the contributing reasons for mobility limitations, with participants experiencing musculoskeletal conditions, neurological conditions, chronic pain, and other conditions that impacted their mobility. Due to the small number of persons in each category, we were unable to explore data by participant social location (e.g., gender, number of daily medications, age).

**Table 1:** Participant numbers and demographics across each step.

Demographics	Brainstorming (n = 22)	Sorting (n = 30)	Rating (n = 45)	Mapping (n = 9)
***Language***				
English	18	27	40	9
French	4	3	5	0
***Age***				
18-29	4	6	8	0
30-39	4	9	10	1
40-49	1	7	10	2
50-59	4	4	9	3
60+	7	4	8	3
Missing	2	0	0	0
***Gender***				
Man	5	4	9	0
Woman	15	24	33	9
Transgender	0	0	0	0
Non-binary	2	2	3	0
Other	0	0	0	0
***Contributing Reasons for Mobility Limitation***				
Musculoskeletal	7	9	14	5
Neurological	6	5	9	0
Chronic pain	3	9	13	3
Other*	2	7	9	1
Missing	4	0	0	0
***Number of Daily Medications***				
0	0	0	0	0
1-4	4	13	18	1
5-9	9	12	16	3
10-14	6	2	7	3
15+	3	3	4	2
Difficulty Walking Long Distance**				
None	0	0	0	0
Mild	3	1	2	0
Moderate	7	14	17	5
Severe	2	7	10	1
Extreme	6	8	16	3
Missing	4	0	0	0

*Other contributing factors of mobility limitations included: a combination of musculoskeletal, neurological and/or chronic pain (e.g., fibromyalgia)

**Over the past 30 days difficulty walking a long distance (e.g., 1 km)

### Cluster map

**Fig 1:**
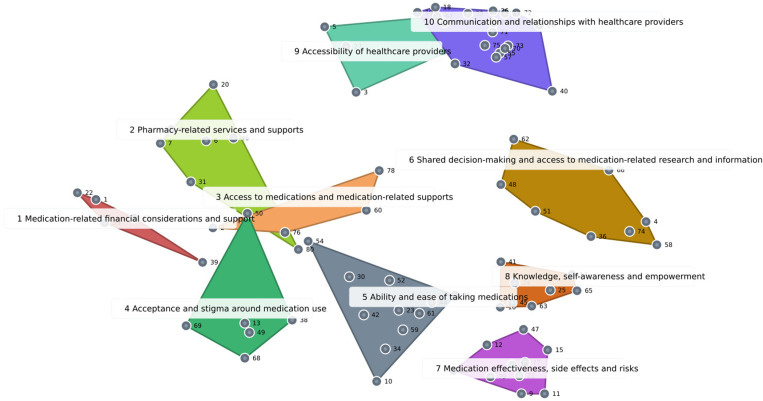
10-Cluster map solution. Legend: Each independent shape represents a cluster with corresponding statements grouped together by participants. Each cluster has a title reflecting the overall concept of the statements assigned. The 10 clusters are the following: Cluster 1 - Medication-related financial considerations and support; Cluster 2 - Pharmacy-related services and supports; Cluster 3 - Access to medications and medication-related supports; Cluster 4 - Acceptance and stigma around medication use; Cluster 5 - Ability and ease of taking medications; Cluster 6 - Shared decision-making and access to medication-related research and information; Cluster 7 - Medication effectiveness, side effects and risks; Cluster 8 - Knowledge, self-awareness and empowerment; Cluster 9 - Accessibility of healthcare providers; Cluster 10 - Communication and relationships with healthcare providers.

Participants selected the 10-cluster map as the final cluster map solution (see Fig 1). The cluster labels included the following: (1) medication-related financial considerations and support; (2) pharmacy-related services and supports; (3) access to medications and medication-related supports; (4) acceptance and stigma around medication use; (5) ability and ease of taking medications; (6) shared decision-making and access to medication-related research and information; (7) medication effectiveness, side effects and risks; (8) knowledge, self-awareness and empowerment; (9) accessibility of healthcare providers; and (10) communication and relationships with healthcare providers.

#### Cluster 1 – Medication-related financial considerations and support.

Cluster 1 contained five statements related to the cost of medications, financial assistance for medications, ability to navigate medication plans, and the impact of medication costs (see Table 2 for clusters and statements). Examples of the statements in this cluster include # 1 – ‘I have access to financial assistance for my medications’, # 43 – ‘I can pay for my medications without negative impacts on my financial well-being’, # 53 – ‘The cost associated with getting my medications is minimal’, and # 39 – ‘Medication plans are user-friendly, consistent, and easy to navigate’.

#### Cluster 2 – Pharmacy-related services and supports.

Cluster 2 contained seven statements related to services and supports offered by the pharmacy team, including providing an emergency supply of medication, completing medication reviews, communicating about medication refills, and adapting prescriptions. Examples of the statements in this cluster include # 6 – ‘Policies to access medications (e.g., insurance plans) have minimal restrictions or requirements’, # 7 – ‘My pharmacist can provide me with an emergency supply of my medications’, # 31 – ‘My pharmacy has my medication in stock’, and # 46 – ‘My pharmacy informs me when my medications are ready (or if they will not be ready on time)’.

#### Cluster 3 – Access to medications and medication-related supports.

Cluster 3 contained four statements related to accessing medications and required tests for starting or continued use (e.g., vaccinations, bloodwork, x-rays), the length of medication supply, and limiting stress associated with accessing medications. Examples of statements include # 76 – ‘I can access the medications that I need without undue stress’, # 78 – ‘I can easily access the diagnostic tests required to ensure my medications are working correctly (e.g., blood work, x-rays)’, and # 60 – ‘My medications are easy to start (e.g., vaccinations, blood work, x-rays)’.

#### Cluster 4 – Acceptance and stigma around medication use.

Cluster 4 contained six statements about being accepted when taking medications, not feeling judged or discriminated against, having support from friends and family, being able to talk to others about medication use, and being able to take medications in public places. Examples of the statements in this cluster include # 50 – ‘I have support from friends and family about my medications’, # 49 – ‘I am not discriminated against by anyone because of my medication use’, and # 69 – ‘My friends and family accept that I take medications’.

#### Cluster 5 – Ability and ease of taking medications.

Cluster 5 contained 11 statements related to starting new medications and continued use of medications, including tests required, traveling with medications, storing medications, having reminders or a routine for taking medications, and independently taking medications. Examples of statements include # 44 – ‘I can take my medications on my own’, # 61 – ‘I can store my medications safely’, # 42 – ‘I can easily travel with my medications’, # 30 – ‘I have enough of my medications to ensure that I never run out (e.g., between refills or renewals)’, and # 35 – ‘I have a routine for taking my medications that works for me’.

#### Cluster 6 – Shared decision-making and access to medication-related research and information.

Cluster 6 contained eight statements related to knowing reliable sources of information, having access to up-to-date medication information from different sources, being actively involved in decisions around medications, and self-advocacy. Example statements include # 36 – ‘I know the difference between reliable and unreliable sources of medication information’, # 48 – ‘I can advocate for myself about my medications and need for any testing (e.g., changes to medications, mode of administration, new medications)’, # 51 – ‘I am informed about my medications (e.g., dosage, timing, why I’m taking it, interactions, side effects)’, and # 62 – ‘I am included in decisions about my medications’.

#### Cluster 7 – Medication effectiveness, side effects and risks.

Cluster 7 contained 10 statements about the effectiveness of medications, side effects of medications including the long-term impact, and medication cascades. Examples of the statements in this cluster include # 9 – ‘My medications will not have negative long-term impacts on me’, # 11 – ‘My medications have minimal withdrawal effects’, # 15 – ‘My medications are effective at controlling my symptoms and managing my condition’, and # 28 – ‘My medications remain effective over time’.

#### Cluster 8 – Knowledge, self-awareness and empowerment.

Cluster 8 contained eight statements related to knowing what medications are taken and what is best, accepting medication use, tracking medications, having a list of medications, supplementing medications with alternatives, and having knowledge around medication doses (e.g., adjusting or missed dose). Examples of statements include # 24 – ‘I know what to do if I miss a dose of my medications’, # 65 – ‘I know what medications are best for me’, # 25 – ‘I know how to adjust my medication doses as needed’, and # 63 – ‘I have a way of tracking the medications that I have taken’.

#### Cluster 9 – Accessibility of healthcare providers.

Cluster 9 contained five statements related to the ability to physically access healthcare providers of one’s choice, meet using different methods, and receive second opinions. Examples of the statements in this cluster include # 67 – ‘I can access my healthcare providers in a timely and convenient manner’, # 27 – ‘My healthcare providers’ locations are accessible and barrier-free’, and # 5 – ‘I have access to the healthcare providers of my choice’.

#### Cluster 10 – Communication and relationships with healthcare providers.

Cluster 10 contained 16 statements about an individuals’ relationship with their healthcare providers, including communication, support received, information sharing, trust, and knowledge. Examples of statements include # 19 – ‘My healthcare providers are knowledgeable about medications’, # 70 – ‘I trust my healthcare providers’, # 64 – ‘My healthcare providers help me start, modify, or stop my medications’, # 40 – ‘I come prepared with a list of questions to appointments with my healthcare providers’, and # 33 – ‘My healthcare providers communicate with each other about my medications (e.g., through an electronic medical record, by fax/telephone)’.

**Table 2:** Cluster names, statements, mean importance, and realistic ratings.

Cluster Name	#	Statements	Mean Importance Rating^a^	Mean Realistic Rating^a^	Importance Rating^b^	Realistic Rating^b^
Cluster 1: Medication-related financial considerations and support	**1**	**I have access to financial assistance for my medications**	**4.64**	**3.62**	**High**	**High**
	22	Medication plans provide financial assistance for over-the-counter medications and natural health products	3.87	2.16	Moderate	Low
	43	I can pay for my medications without negative impacts on my financial well-being	4.49	2.76	High	Moderate
	53	The cost associated with getting my medications is minimal	4.53	2.78	High	Moderate
	39	Medication plans are user-friendly, consistent, and easy to navigate	4.09	3.07	Moderate	Moderate
		Mean Cluster Score	4.32	2.88	High	Moderate
Cluster 2: Pharmacy-related services and supports	6	Policies to access medications (e.g., insurance plans) have minimal restrictions or requirements	4.27	2.78	High	Moderate
	**7**	**My pharmacist can provide me with an emergency supply of my medications**	**4.56**	**3.67**	**High**	**High**
	8	My pharmacist can adapt my prescriptions (e.g., mode of administration, brand)	3.8	3.38	Moderate	Moderate
	20	I can meet with my pharmacist to review my medications	3.8	3.98	Moderate	High
	**31**	**My pharmacy has my medications in stock**	**4.33**	**3.73**	**High**	**High**
	**46**	**My pharmacy informs me when my medications are ready (or if they will not be ready on time)**	**4.31**	**4.11**	**High**	**High**
	80	I can get the medications that I prefer (e.g., name brand, generic)	3.78	3.42	Moderate	Moderate
		Mean Cluster Score	4.12	3.58	Moderate	Moderate
Cluster 3: Access to medications and medication-related supports	2	I can get a three-month supply of my medications to minimize my need to return to the pharmacy	3.93	3.69	Moderate	High
	60	My medications are easy to start (e.g., vaccinations, blood work, x-rays)	3.93	3.76	Moderate	High
	76	I can access the medications that I need without undue stress	4.42	3.47	High	Moderate
	78	I can easily access the diagnostic tests required to ensure my medications are working correctly (e.g., blood work, x-rays)	4.22	3.42	High	Moderate
		Mean Cluster Score	4.13	3.59	Moderate	Moderate
Cluster 4: Acceptance and stigma around medication use	13	I do not feel judged when taking my medications (e.g., in public places, social situations)	3.11	3.42	Low	Moderate
	38	I can talk with others who have experience with my medications	3.73	3.2	Moderate	Moderate
	49	I am not discriminated against by anyone because of my medication use	4.13	3.76	Moderate	High
	68	Accommodations are made for me to take my medications in public places	3.33	3.18	Moderate	Moderate
	69	My friends and family accept that I take medications	3.96	4.04	Moderate	High
	**50**	**I have support from friends and family about my medications**	**4.16**	**3.93**	**High**	**High**
		Mean Cluster Score	3.74	3.59	Moderate	Moderate
Cluster 5: Ability and ease of taking medications	10	My medications require minimal tests (e.g., x-ray, MRI) for starting and/or ongoing use	3.8	3.27	Moderate	Moderate
	**23**	**I can bring my medications with me when I leave my home**	**4.31**	**4.24**	**High**	**High**
	**30**	**I have enough of my medications to ensure that I never run out (e.g., between refills or renewals)**	**4.42**	**3.78**	**High**	**High**
	34	The number of medications I have to take is minimal	3.64	2.8	Moderate	Moderate
	**35**	**I have a routine for taking my medications that works for me**	**4.42**	**4.16**	**High**	**High**
	**42**	**I can easily travel with my medications**	**4.44**	**3.93**	**High**	**High**
	**44**	**I can take my medications on my own**	**4.67**	**4.58**	**High**	**High**
	**52**	**My medications are easy to take**	**4.38**	**4.11**	**High**	**High**
	59	I have reminders for taking my medications	3.67	3.71	Moderate	High
	**61**	**I can store my medications safely**	**4.44**	**4.33**	**High**	**High**
	54	My medications and medical appointments do not interfere with activities that matter to me	3.91	2.84	Moderate	Moderate
		Mean Cluster Score	4.19	3.80	High	High
Cluster 6: Shared decision-making and access to medication-related research and information	4	I stay up to date with new medications that have been approved for my condition	3.82	3.67	Moderate	High
	**36**	**I know the difference between reliable and unreliable sources of medication information**	**4.47**	**4.27**	**High**	**High**
	**48**	**I can advocate for myself about my medications and need for any testing (e.g., changes to medications, mode of administration, new medications)**	**4.44**	**4.11**	**High**	**High**
	**51**	**I am informed about my medications (e.g., dosage, timing, why I’m taking it, interactions, side effects)**	**4.62**	**4.16**	**High**	**High**
	**58**	**Studies have been conducted about my medications (e.g., on the risks and benefits)**	**4.36**	**3.98**	**High**	**High**
	**62**	**I am included in decisions about my medications**	**4.38**	**4.07**	**High**	**High**
	66	I can test medications to see what works best for me (trialing medications or doses)	4.11	3.53	Moderate	Moderate
	**74**	**I can access information about my medications from multiple sources on my own**	**4.2**	**4.16**	**High**	**High**
		Mean Cluster Score	4.30	3.99	High	High
Cluster 7: Medication effectiveness, side effects and risks	9	My medications will not have negative long-term impacts on me	4.31	2.69	High	Moderate
	11	My medications have minimal withdrawal effects	4.33	3.24	High	Moderate
	12	I keep a diary of how I am feeling with my medications (e.g., symptoms, side effects, questions)	2.71	2.71	Low	Moderate
	15	My medications are effective at controlling my symptoms and managing my condition	4.56	3.44	High	Moderate
	21	My medications have few and/or tolerable side effects	4.33	3.24	High	Moderate
	28	My medications remain effective over time	4.36	3.44	High	Moderate
	47	My medications make me feel like myself	4.2	3.36	High	Moderate
	56	I know the medicinal and non-medicinal ingredients in my medications	3.44	2.69	Moderate	Moderate
	**77**	**The benefits of my medications outweigh any negative effects**	**4.44**	**3.84**	**High**	**High**
	79	I am not taking medications to treat side effects of other medications	4.07	3.47	Moderate	Moderate
		Mean Cluster Score	4.08	3.21	Moderate	Moderate
Cluster 8: Knowledge, self-awareness and empowerment	14	I do not feel overwhelmed because of my medications	3.76	3.36	Moderate	Moderate
	16	I always keep my medication list with me	3.64	3.6	Moderate	High
	**24**	**I know what to do if I miss a dose of my medications**	**4.33**	**4.42**	**High**	**High**
	25	I know how to adjust my medication doses as needed	4.07	3.89	Moderate	High
	41	I can supplement my medications with lifestyle changes or other therapies (e.g., diet, exercise, physiotherapy, counselling)	3.91	3.51	Moderate	Moderate
	**45**	**I accept that I need to take medications**	**4.51**	**4.6**	**High**	**High**
	**63**	**I have a way of tracking the medications that I have taken**	**4.18**	**3.93**	**High**	**High**
	65	I know what medications are best for me	4.31	3.51	High	Moderate
		Mean Cluster Score	4.09	3.85	Moderate	High
Cluster 9: Accessibility of healthcare providers	3	I can get a second opinion about my medications	3.71	3.27	Moderate	Moderate
	5	I have access to the healthcare providers of my choice	4.22	2.87	High	Moderate
	**27**	**My healthcare providers’ locations are accessible and barrier-free**	**4.29**	**3.6**	**High**	**High**
	67	I can access my healthcare providers in a timely and convenient manner (e.g., no waiting, same day appointments, extended hours, conveniently located)	4.49	2.64	High	Moderate
	71	I can meet with my healthcare providers through different forums depending on my needs and preferences (e.g., in-person, telephone, video-conferencing, email)	4.13	3.64	Moderate	High
		Mean Cluster Score	4.17	3.35	High	Moderate
Cluster 10: Communication and relationships with healthcare providers	**17**	**I have a good relationship with my healthcare providers (e.g., positive interactions)**	**4.38**	**3.93**	**High**	**High**
	18	My healthcare providers do not give contradictory information about my medications	4.2	3.51	High	Moderate
	**19**	**My healthcare providers are knowledgeable about medications**	**4.64**	**4.09**	**High**	**High**
	**26**	**My healthcare providers consider me when recommending medications (e.g., my values, preferences, independence, ability to pay, ability to take)**	**4.27**	**3.8**	**High**	**High**
	29	I am supported by my healthcare providers with medication testing (trial and error) to see what works for me	4.22	3.58	High	Moderate
	32	My healthcare providers help me integrate medication taking into my everyday life	3.87	3.53	Moderate	Moderate
	33	My healthcare providers communicate with each other about my medications (e.g., through an electronic medical record, by fax/telephone)	4.24	3.24	High	Moderate
	**37**	**My healthcare providers make sure I understand the medication information that they share with me (e.g., ask me to repeat it back to them)**	**4.2**	**3.71**	**High**	**High**
	55	My healthcare providers have enough time to spend with me to discuss my medications	4.27	3.18	High	Moderate
	**57**	**My healthcare providers are receptive to my thoughts on my medications**	**4.27**	**3.62**	**High**	**High**
	**64**	**My healthcare providers help me start, modify, or stop my medications**	**4.33**	**4.09**	**High**	**High**
	**70**	**I trust my healthcare providers**	**4.58**	**4.02**	**High**	**High**
	72	My healthcare providers are willing to talk to me about cannabis for medicinal use	3.2	3.47	Moderate	Moderate
	**73**	**My healthcare providers believe me when I tell them my medications are not adequately addressing my symptoms**	**4.44**	**3.76**	**High**	**High**
	75	My healthcare providers follow up with me about my medications	3.96	3.6	Moderate	High
	**40**	**I come prepared with a list of questions to appointments with my healthcare providers**	**4.16**	**4.18**	**High**	**High**
		Mean Cluster Score	4.20	3.71	High	High

a Rating scale: 1 = not at all important/realistic; 2 = slightly important/realistic; 3 = moderately important/realistic; 4 = very important/realistic; 5 = extremely important/realistic.

b Mean ratings are considered high in the ‘Go-Zone’ if above the mean score for all the statements rated on importance (mean 4.14) and realistic (mean 3.59), moderate if >1 point below the mean, and low if <1 point below the mean, respectively. Bolded statements met the criteria threshold for the ‘Go-Zone’.

### Cluster and statement ratings

**Fig 2:**
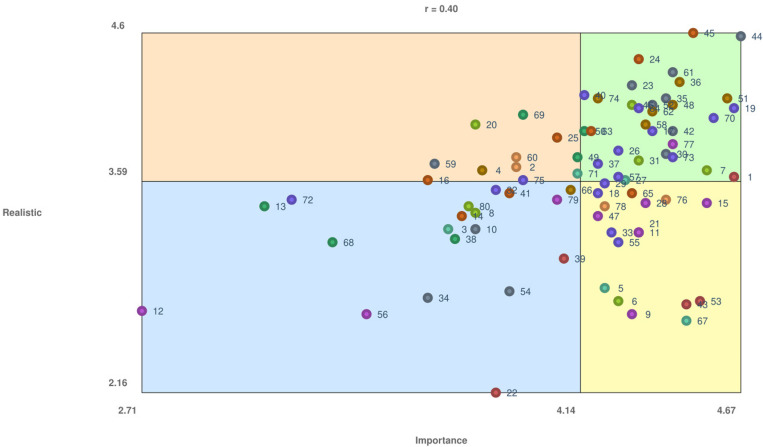
Go-Zone Plot. Legend: The x axis shows the ratings of importance on the 80 statements and the y axis shows the ratings of how realistic to address the 80 statements. The go-zone (green box, upper right quadrant) included 32 of the 80 statements. The r-value was 0.40, reflecting a moderate correlation between ratings for importance and realistic. The statement rated highest on both importance and realistic was *44 - I can take my medications on my own* (mean = 4.67; 4.58, respectively). Cluster 10 - Communication and relationships with healthcare providers contained the most statements within the go-zone, with a total of nine statements.

Overall, the go-zone included 32 of the 80 statements (see Fig 2). With an r-value of 0.40, there was a moderate association between ratings for importance and realistic. Cluster 1 – ‘Medication-related financial considerations and supports’ was rated the highest on importance (mean = 4.32) and the lowest on realistic to act on (mean = 2.88). The second most important cluster was Cluster 6 – ‘Shared decision-making and access to medication-related research and information’ (mean = 4.30) and the highest on realistic to act on (mean = 3.99).

## Discussion

Using a concept mapping approach, we engaged with persons who have lived experience with mobility limitations to identify what matters about their medications in everyday life. Concept mapping is a participatory method that fosters shared decision-making throughout the various research stages. Participants identified a total of 80 unique statements, which mapped onto ten clusters noted to be relevant to persons with mobility limitations taking medications. The top rated clusters on importance were mostly related to finances, shared-decision making, communication and accessibility of healthcare providers, and ability/ease in taking medications.

Many of the clusters identified in our study map onto those identified within the general population as described by Katusiime and colleagues [37]. In their systematic review, Katusiime and colleagues (2016) identified and compared generic PREMs for prescribed medications. In addition, the authors collated a list of domains being captured in current PREM studies, which included the following: effectiveness; convenience, practicalities, and/or managing medications; information, knowledge, and/or understanding; side-effects; relationships and/or communication with health professionals; impact on daily living and/or social life; general satisfaction; attitudes; beliefs, concerns, and/or perceptions; medical follow-up, and/or adherence-related issues; treatment and/or medicine related burden, perceived control, or autonomy; self-confidence about medication use; availability and accessibility; and medicine-related quality of life.

Interestingly, there are a few measures for the general population that represent most of the domains identified in our study, such as the 43-item Patient Reported Outcomes Measure of a Pharmaceutical Therapy for Quality of Life (PROMPT-QoL) [38] and the 41-item Living with Medicines Questionnaire (LMQ-3) [39]. However, our study has a few unique considerations for persons with mobility limitations that do not seem to exist in current generic measures. The unique statements identified in our study relate to costs and financial assistance, access and availability of healthcare providers and medications (e.g., refills, medications in stock, emergency supply), ability and ease of physically taking medications (e.g., autonomy in taking medications on own), and communication and relationships with healthcare providers. Further, in our study, a separate cluster was dedicated to medication-related financial assistance and support, with the statement on access to financial assistance for medications being rated as the most important to participants. In contrast, PROMPT-QoL does not have any explicit statements about financial assistance and/or related burden [38]. The LMQ-2 was recently updated to the LMQ-3 to capture cost-related concerns for medications, with three items specifically related to cost-related burden [39]. Importantly, persons with disabilities often have lower income [40], which may impact their ability to access and use medications as well as their overall experiences [24,41–43]. For example, Gupta and colleagues explored medication cost burden among persons with spinal cord injury in Canada, and noted many participants were not able to take medications as prescribed due to costs [41]. Similarly, a national survey from Statistics Canada identified costs as a major contributor to unmet needs for persons with disabilities, with 13% of all persons with disabilities aged 15 years and over having unmet needs for prescription medications [44]. In addition to costs, participants in our study identified physical ability or supports in taking medications as important. This was illustrated with statement # 44 - ‘I can take my medications on my own’*.* However, neither the PROMPT-QoL or the LMQ-3 address this domain. Instead, the PROMPT-QoL has a statement related to “convenience of use” [38] and the LMQ-3 has a generic item related to use, “I find using my medicines difficult” [39].

Our study also reinforced the importance of communication, positive relationships, and shared decision-making with many types of healthcare providers (i.e., including but beyond physicians). Most of the statements related to these two key clusters (Clusters 6 and 10) were within the go-zone, and as such, were rated high on importance and realistic to address. These statements related to overall positive interactions with healthcare providers, such as fostering trust and discussing concerns without being rushed. To address health system capacity challenges, many jurisdictions have broadened the health professionals involved in prescribing and medication management to include pharmacists, nurse practitioners, and nurses. Relatedly, it is important for PREMs to measure experiences with a diverse range of providers to reflect these changes in healthcare delivery. As an example of current PREM limitations, the LMQ-3 [39] is physician-centric in wording, with a focus on patient-physician relationships rather than a more inclusive array of healthcare professionals who may prescribe and/or be involved in medication therapy management.

This study has a few limitations. First, we experienced challenges with recruitment during the COVID-19 pandemic. The relatively smaller sample size, while adequate for concept mapping, limited our abilities to conduct sub-analyses to explore how social location may influence what matters to persons with mobility limitations about their medications in everyday life. Relatedly, we had hoped for more participants who were French speaking (20% of our participants). To reduce burden, we adjusted the French language data collection process to be conducted with a research team member instead of in an online group or asynchronously. Despite significant recruitment strategies and extending recruitment time, we were unable to increase our participation. We suspect this was largely due to the pandemic. Despite these challenges, there are several strengths to this study. To our knowledge, this is the first study that has examined, in a participatory manner using concept mapping, what matters to persons with mobility limitations about their medications. We identified comprehensive ideas through numerous virtual engagement activities. While virtual data collection may have limited participation among those with limited access to the internet/telephone, we consider this method a strength in being more inclusive to persons with mobility limitations across Canada by allowing participation with reduced physical barriers. Persons with disabilities have often experienced systemic exclusion in society and this study helps contribute to elevating what matters for this population.

There are several gaps that remain in existing PREMs to address the areas identified as important to persons with mobility limitations and the general population more broadly [45]. Measuring and optimizing patient experiences are of critical importance to value-based quality care [10]. Despite the high use of prescribed medications in Canada and globally [46,47], PREMs for medications are not used in routine clinical practice [45]. Based on this study’s findings, a separate PREM does not seem to be warranted for persons with mobility limitations, but rather, modifying or supplementing existing measures to capture concepts relevant to this population (e.g., financial assistance, access, communication, and relationship with an array of healthcare providers involved in medication management) is necessary. At the patient level, integrating a PREM into routine practice might facilitate person-centred shared decision-making in understanding what matters most to individuals, potentially increasing patient satisfaction and care quality. At the healthcare provider level, implementing a PREM into practice might guide consultations about medications in a standardized way and help optimize providers’ full scope of practice in person-centred care. Finally, at the health system level, a PREM might provide additional metrics for person-centred care that could be built into existing remuneration models for medication reviews; thus, further optimizing overall medication management. Identifying a core set of items and understanding implementation considerations would be an important next step from the existing literature and should involve key experts and interest groups (e.g., persons with lived experience, prescribers and other healthcare providers, decision-makers, administrators).

## Conclusions

There are currently no PREMs or PROMs that apply a comprehensive framework on the experiences with or impact of medications on everyday life for persons with mobility limitations. This research has identified key items and domains related to medication-related experiences for persons with mobility limitations that will inform the development of a responsive and meaningful PREM in efforts to achieve improved healthcare experiences for Canadian adults who take medications.

## Supporting information

S1 Fig Concept mapping point map. The total square similarity matrix was the input for multidimensional scaling, producing the point map.
(TIF)

S1 Table Go zone statements, clusters, and mean ratings. Rating scale: 1 = not at all important/realistic; 2 = slightly important/realistic; 3 = moderately important/realistic; 4 = very important/realistic; 5 = extremely important/realistic.
(DOCX)
