# Supplementary figures and images for: Identifying what matters to adults with mobility limitations regarding their experiences with medications: A concept mapping study
Source: PLoS One. 2025 May 23;20(5):e0323877. doi: 10.1371/journal.pone.0323877 (PMC12101783; doi:10.1371/journal.pone.0323877)

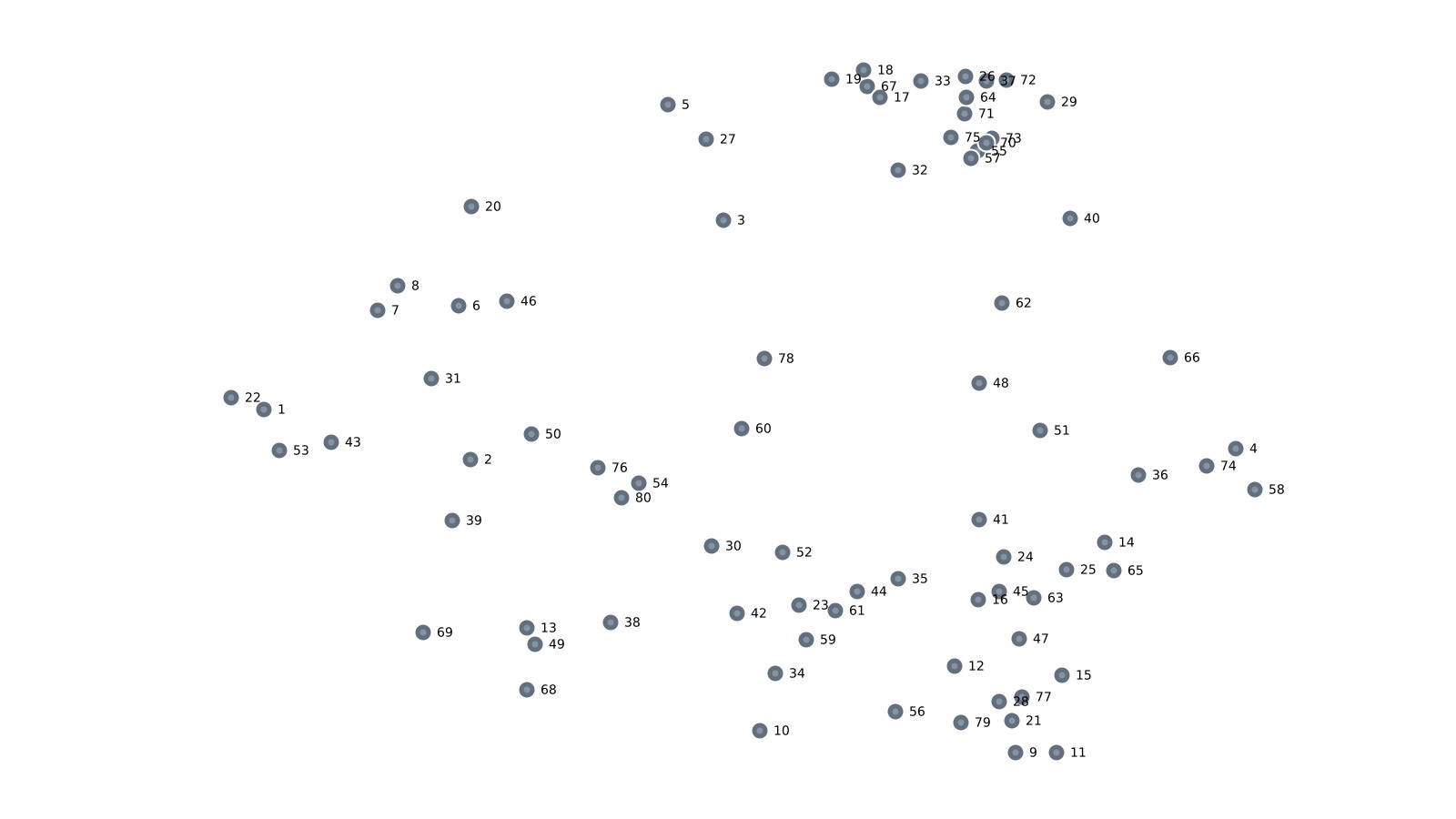

Supplement: S1 Fig — The total square similarity matrix was the input for multidimensional scaling, producing the point map. (TIF) [file pone.0323877.s001.tif]
