# Supplementary material for: Identifying what matters to adults with mobility limitations regarding their experiences with medications: A concept mapping study
Source: PLoS One. 2025 May 23;20(5):e0323877. doi: 10.1371/journal.pone.0323877 (PMC12101783; doi:10.1371/journal.pone.0323877)
Supplement: S1 Table — Rating scale: 1 = not at all important/realistic; 2 = slightly important/realistic; 3 = moderately important/realistic; 4 = very important/realistic; 5 = extremely important/realistic. (DOCX) [file pone.0323877.s002.docx]

**Supplementary Table 1. Go zone statements, clusters, and mean ratings**

| **#** | **Go Zone Statements** | **Cluster Number and Title** | **Mean Importance Rating^a^** | **Mean Realistic Rating^a^** |
| --- | --- | --- | --- | --- |
| 1 | I have access to financial assistance for my medications | CLUSTER 1 - Medication-related financial considerations and support | 4.64 | 3.62 |
| 7 | My pharmacist can provide me with an emergency supply of my medications | CLUSTER 2 - Pharmacy-related services and supports | 4.56 | 3.67 |
| 17 | I have a good relationship with my healthcare providers (e.g., positive interactions) | CLUSTER 10 - Communication and relationships with healthcare providers | 4.38 | 3.93 |
| 19 | My healthcare providers are knowledgeable about medications | CLUSTER 10 - Communication and relationships with healthcare providers | 4.64 | 4.09 |
| 23 | I can bring my medications with me when I leave my home | CLUSTER 5 - Ability and ease of taking medications | 4.31 | 4.24 |
| 24 | I know what to do if I miss a dose of my medications | CLUSTER 8 - Knowledge, self-awareness and empowerment | 4.33 | 4.42 |
| 26 | My healthcare providers consider me when recommending medications (e.g., my values, preferences, independence, ability to pay, ability to take) | CLUSTER 10 - Communication and relationships with healthcare providers | 4.27 | 3.80 |
| 27 | My healthcare providers' locations are accessible and barrier-free | CLUSTER 9 - Accessibility of healthcare providers | 4.29 | 3.60 |
| 30 | I have enough of my medications to ensure that I never run out (e.g., between refills or renewals) | CLUSTER 5 - Ability and ease of taking medications | 4.42 | 3.78 |
| 31 | My pharmacy has my medications in stock | CLUSTER 2 - Pharmacy-related services and supports | 4.33 | 3.73 |
| 35 | I have a routine for taking my medications that works for me | CLUSTER 5 - Ability and ease of taking medications | 4.42 | 4.16 |
| 36 | I know the difference between reliable and unreliable sources of medication information | CLUSTER 6 - Shared decision-making and access to medication-related research and information | 4.47 | 4.27 |
| 37 | My healthcare providers make sure I understand the medication information that they share with me (e.g., ask me to repeat it back to them) | CLUSTER 10 - Communication and relationships with healthcare providers | 4.20 | 3.71 |
| 40 | I come prepared with a list of questions to appointments with my healthcare providers | CLUSTER 10 - Communication and relationships with healthcare providers | 4.16 | 4.18 |
| 42 | I can easily travel with my medications | CLUSTER 5 - Ability and ease of taking medications | 4.44 | 3.93 |
| 44 | I can take my medications on my own | CLUSTER 5 - Ability and ease of taking medications | 4.67 | 4.58 |
| 45 | I accept that I need to take medications | CLUSTER 8 - Knowledge, self-awareness and empowerment | 4.51 | 4.60 |
| 46 | My pharmacy informs me when my medications are ready (or if they will not be ready on time) | CLUSTER 2 - Pharmacy-related services and supports | 4.31 | 4.11 |
| 48 | I can advocate for myself about my medications and need for any testing (e.g., changes to medications, mode of administration, new medications) | CLUSTER 6 - Shared decision-making and access to medication-related research and information | 4.44 | 4.11 |
| 50 | I have support from friends and family about my medications | CLUSTER 4 - Acceptance and stigma around medication use | 4.16 | 3.93 |
| 51 | I am informed about my medications (e.g., dosage, timing, why I'm taking it, interactions, side effects) | CLUSTER 6 - Shared decision-making and access to medication-related research and information | 4.62 | 4.16 |
| 52 | My medications are easy to take | CLUSTER 5 - Ability and ease of taking medications | 4.38 | 4.11 |
| 57 | My healthcare providers are receptive to my thoughts on my medications | CLUSTER 10 - Communication and relationships with healthcare providers | 4.27 | 3.62 |
| 58 | Studies have been conducted about my medications (e.g., on the risks and benefits) | CLUSTER 6 - Shared decision-making and access to medication-related research and information | 4.36 | 3.98 |
| 61 | I can store my medications safely | CLUSTER 5 - Ability and ease of taking medications | 4.44 | 4.33 |
| 62 | I am included in decisions about my medications | CLUSTER 6 - Shared decision-making and access to medication-related research and information | 4.38 | 4.07 |
| 63 | I have a way of tracking the medications that I have taken | CLUSTER 8 - Knowledge, self-awareness and empowerment | 4.18 | 3.93 |
| 64 | My healthcare providers help me start, modify, or stop my medications | CLUSTER 10 - Communication and relationships with healthcare providers | 4.33 | 4.09 |
| 70 | I trust my healthcare providers | CLUSTER 10 - Communication and relationships with healthcare providers | 4.58 | 4.02 |
| 73 | My healthcare providers believe me when I tell them my medications are not adequately addressing my symptoms | CLUSTER 10 - Communication and relationships with healthcare providers | 4.44 | 3.76 |
| 74 | I can access information about my medications from multiple sources on my own | CLUSTER 6 - Shared decision-making and access to medication-related research and information | 4.20 | 4.16 |
| 77 | The benefits of my medications outweigh any negative effects | CLUSTER 7 - Medication effectiveness, side effects and risks | 4.44 | 3.84 |

^a^Rating scale: 1= not at all important/realistic; 2= slightly important/realistic; 3= moderately important/realistic; 4= very important/realistic; 5= extremely important/realistic.
